# Diagnostic usefulness of pregnancy-associated plasma protein-A in suspected pulmonary embolism

**DOI:** 10.1186/2049-6958-8-49

**Published:** 2013-07-31

**Authors:** Serdar Berk, Omer Tamer Dogan, Eylem Itir Aydemir, Asli Bingol, Sefa Levent Ozsahin, Ibrahim Akkurt

**Affiliations:** 1 Department of Chest Diseases, Medical Faculty of Cumhuriyet University, Sivas, Turkey; 2 Deparment of Statistics, Science Faculty of Cumhuriyet University, Sivas, Turkey

**Keywords:** Inflammation, Pulmonary embolism, PAPP-A

## Abstract

**Background:**

The role of biomarkers for prognostication and diagnosis of pulmonary embolism (PE) is increasing. It has been reported that pregnancy-associated plasma protein-A (PAPP-A) can be used as a proatherosclerotic marker. The present study was aimed to evaluate whether PAPP-A levels are helpful in the differential diagnosis of patients presenting with suspected PE.

**Methods:**

53 consecutive patients evaluated for suspected PE were prospectively enrolled in the study. Serum PAPP-A levels were measured in the blood samples which were taken at admission. Multi-slice computed tomographic angiography was used to verify the diagnosis of PE.

**Results:**

PE was detected in 24 out of the 53 patients, while it was excluded in 29 patients by thorax multi-detector computerized tomography scan. No significant difference was detected in mean serum PAPP-A level between groups (5.72 ± 0.31 mg/L vs. 5.67 ± 0.06 mg/L, respectively).

**Conclusions:**

Serum PAPP-A level has no role in the evaluation for PE.

## Background

Pulmonary embolism (PE) is an important clinical entity with high mortality and morbidity. Particularly, mortality is determined by hemodynamic status and underlying disease. The role of biomarkers for the prognostication and diagnosis of PE is increasing [1,2].

The pregnancy-associated plasma protein-A (PAPP-A) is a matrix metalloproteinase which is an activator of insulin-like growth factor-1. The PAPP-A with elevating serum levels by the progression of pregnancy is mainly used as a part of screening tests for Down syndrome [3]. Moreover, it has been reported that its serum level is increased during plaque rupture process in acute coronary syndromes, suggesting that it can be used as a proatherosclerotic marker [4]. The present study was aimed to evaluate whether PAPP-A levels are helpful in the differential diagnosis of patients presented with suspected PE.

## Methods

Overall, 53 consecutive patients presenting to our hospital in the last six months with suspected PE were prospectively enrolled to the study. The study was approved by Local Ethic Committee of the University. All patients gave written informed consent. Demographic characteristics and clinical findings were recorded. The routine laboratory (*e.g.* complete blood count, liver and kidney function tests, D-dimer and Troponin I measurements, arterial blood gas analysis and ECG) and radiological evaluations (e.g. chest X-ray and thoracic CT scan) were performed to establish or exclude the diagnosis of PE.

Besides, routine laboratory evaluations were performed to establish or exclude the diagnosis of PE, and multi-slice thorax CT angiography was performed for definitive diagnosis. All CT evaluations were performed by using 16-slice MDCT scanner (Brilliance iCT, Philips Healthcare, Cleveland OH, USA) and automatic intravenous contrast material injections were performed by MEDRAD's Stellant Injection Systems (Stellant, Medrad, Indianola, USA).

Scanning was performed while patients were in a supine position with both arms being next to head, and lasted approximately 10 seconds. The following acquisition parameters were used: 120 kV; 200 mAs; collimation 16×0.75; pitch value 0.9; and gantry rotation time 0.5. A scanogram was obtained that primarily involves thoracic outlet and diaphragm. The area from diaphragm to aortic arc was identified as region of interest. A tracker was placed at pulmonary trunk to automatically trigger acquisition when contrast material reached to 120 HU within pulmonary artery. First, the area between aortic arc and diaphragm was scanned with a slice thickness of 2 mm. Then, the area between aortic arc and thoracic outlet was scanned with a slice thickness of 0.5 mm. Non-ionic iodine (350 mg/mL) was used as contrast material. Axial, coronal and sagittal planes were assessed by volume rendering Maximum Intensity Projection (MIP) and Multiplanar Reformation (MPR) techniques. In these assessments, standard mediastinal (350/40) and pulmonary (-700/1000) windows were used. The images of multi-slice CT angiography were assessed for presence of embolism in main, lobar, segmental and subsegmental arteries and for sufficiency of vascular contrast enhancement.

Blood samples (5 ml) were drawn into plain tubes to measure PAPP-A levels at presentation. Than samples were centrifuged and sera obtained were stored at -80°C until assays. Before analysis, sera awaited at room temperature. PAPP-A levels were measured by EIA method using ultrasensitive ELISA kit (ELIZEN PAPP-A; Zentech, Angleur, Belgium). Pregnant women, and those with prerenal azotemia, renal failure, liver disease, diabetes, known hypertension and heart diseases were excluded.

### Statistical analysis

The descriptive statistics (mean, standard deviation, frequency) as well as Chi-square and Student’s t tests were used in the data analysis.

## Results

**Figure 1:**
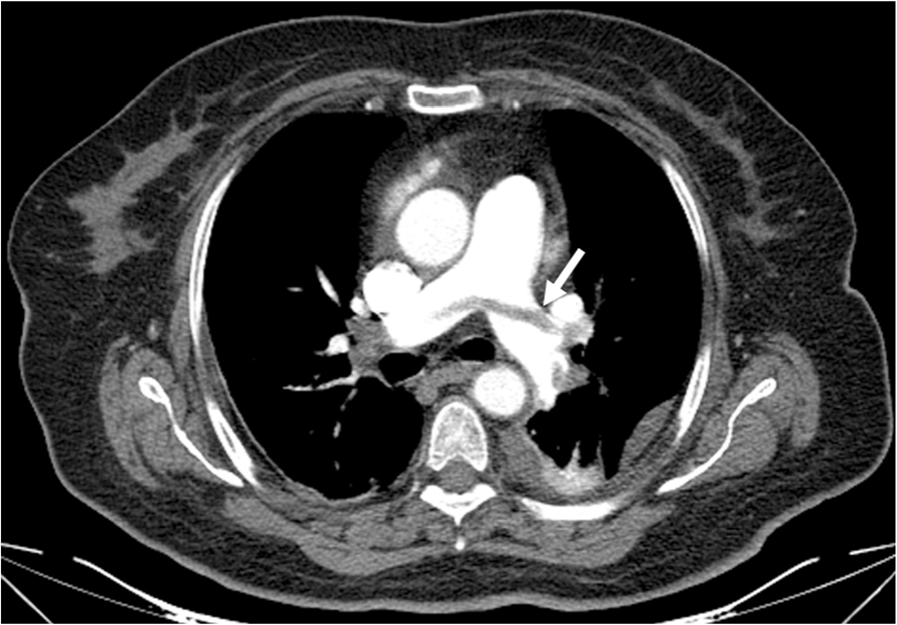
Sixty eight-year-old female patient with pulmonary emboli.

**Figure 2:**
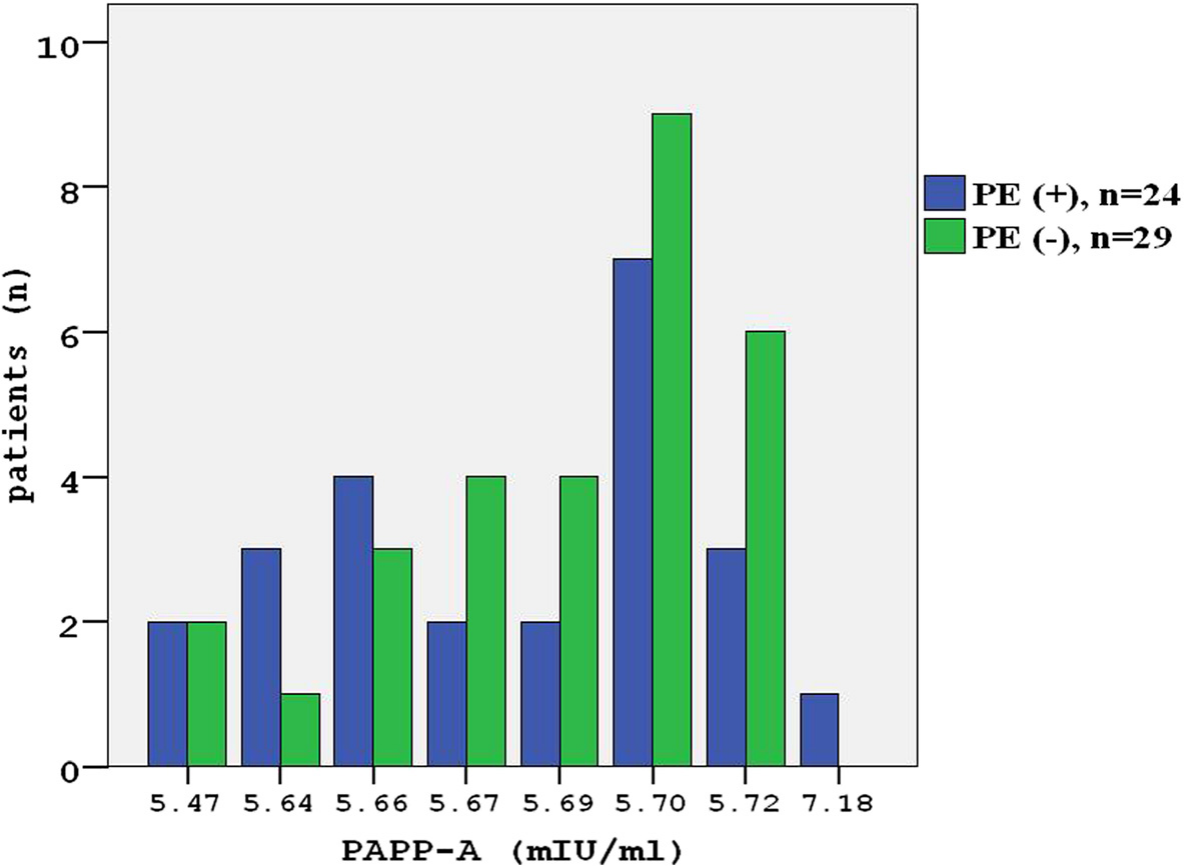
Distribution of serum PAPP-A values of two groups.

On the thorax multi-slice CT angiography, PE was detected in 24 patients (PE+) (Figure 1), while it was excluded in 29 (PE-) of 53 patients. Out of the 29 patients without PE, 20were affected with pneumonia, 5 with COPD exacerbation, one with Dressler syndrome and onewith empyema, while restrictive pulmonary disorders causing acute respiratory stress was detected in two patients. Mean age was 62 ± 17 years in 24 patients with PE, and 63 ± 15 years in 29 patients without PE. 48% of the patients with PE were women, whereas 52% of those without PE were women. The mean age and sex distribution were similar in both groups (p > 0.05). While cough was more frequent in patients without PE, other clinical, radiological and laboratory finding were found to be similar in both groups (Tables 1 and 2). The distribution of serum PAPP-A values was similar in both groups (Figure 2). No significant difference was found in mean serum PAPP-A levels between groups (5.72 ± 0.31 mg/L vs. 5.67 ± 0.06 mg/L, respectively; p = 0.399).

**Table 1:** Comparison of clinical and radiological findings of the study groups

**Parameters**	**PE(+) (n = 24)**	**PE(-) (n = 29)**	**p**
	**n (%)**	**n (%)**	
**Sex (men)**	13 (54)	14(48)	0.669
**Symptoms**			
Shortness of breath	18 (75)	25 (86)	0.299
Chest pain	14 (58)	25 (86)	0.098
Palpitation	12 (50)	10 (34)	0.254
Cough	11 (45)	21 (72)	0.049^*^
Leg swelling	8 (33)	9 (31)	0.858
Hemoptysis	5 (20)	3 (10)	0.288
**Physical examination findings**			
Crackles	14 (58)	22 (76)	0.174
Ronchus	4 (17)	7 (24)	0.504
Cyanosis	6 (25)	13 (44)	0.134
Tachycardia	5 (20)	6 (20)	0.990
Tachypnea	4 (17)	10 (34)	0.143
**Radiological findings**			
Infiltration	14 (58)	22 (76)	0.174
Cardiomegaly	16 (66)	14 (48)	0.179
Linear atelectasis	7 (29)	9 (31)	0.883
Pleural effusion	5 (20)	9 (31)	0.402
Elevation of the Diaphragm	3 (12)	4 (14)	0.890

*p < 0.05 significant difference.

*PE*: Pulmonary embolism.

**Table 2:** Comparison of laboratory features of the study groups

**Parameters**	**PE(+)**	**PE(-)**	**p**
	**Mean ± SD**	**Mean ± SD**	
Age (year)	62 ± 17	63 ± 15	0.720
BMI (kg/m^2^)	28 ± 4	27 ± 3	0.308
Hearth rate (breaths/min)	88 ± 15	92 ± 20	0.511
SBP (mmHg)	121 ± 30	126 ± 29	0.515
DBP (mmHg)	73 ± 17	75 ± 12	0.504
Respiratory rate (breaths/min)	23 ± 3	26 ± 4	0.021^*^
Body temperature	36.7 ± 0.6	36.5 ± 0.8	0.546
pH	7.43 ± 0.06	7.42 ± 0.07	0.675
PaO_2_(mmHg)	55.6 ± 15.2	61.8 ± 18.1	0.286
PaCO_2_(mmHg)	37.3 ± 6.3	36.5 ± 7.6	0.680
SaO_2_ (%)	87.0 ± 7.5	89.8 ± 6.6	0.222
CRP (mg/L)	57 ± 62	81 ± 92	0.293
WBC (x10^3^/μL)	11.6 ± 5.6	15.3 ± 14.2	0.239
Hb (g/dl)	13.3 ± 2.6	13.6 ± 2.3	0.678
Ht (%)	40.8 ± 8.3	40.3 ± 6.6	0.396
PLT (x10^3^)	266 ± 105	260 ± 107	0.865
D-Dimer (ng/ml)	2938 ± 3471	1968 ± 3106	0.313
PAPP-A (mIU/ml)	5.72 ± 0.31	5.67 ± 0.06	0.399

*BMI*: Body mass index, *CRP*: C-reactive protein, *DBP*: Diastolic blood pressure, *Hb*: Hemoglobin, *Ht*: Hematocrit, *PLT*: Platelet, *PAPP-A*: Pregnancy-associated plasma protein-A, *SBP*: Systolic blood pressure, *SD*: Standard deviation, *WBC*: White blood cells.

*p < 0.05 significant difference.

## Discussion

Pulmonary embolism is characterized by the formation of a blood clot in a vein, which will begin to travel through the circulatory system and will become lodged within the pulmonary arterial system. Clinics, radiology, and laboratory findings in patients with PE are associated with the degree of occlusion of arteries, and the cardiopulmonary reserve of the patient. Dyspnoea, pleuritic chest pain, cough, haemoptysis, mild fever, tachypnea, tachycardia, and crackles are the most common symptoms and signs in patients with PE. Atelectasis, pleural effusion, pulmonary infiltrates, and mild elevation of a hemi diaphragm may be seen on chest X-ray. Elevated D-dimer, leukocytosis, hypoxemia may be seen [5]. These findings are nonspecific and can be seen in pneumonia, pleurisy, acute exacerbation of chronic lung disease, acute coronary syndromes, acute congestive heart failure or pulmonary oedema, pneumothorax, and dissecting or rupturing aortic aneurysm [6]. Richman et al. reported 81% pneumonia, 7% aortic aneurysm or dissection, and 7% mass suggesting undiagnosed malignancy were found in patients whom PE were ruled out by chest computed tomography angiography, in emergency departments patients with symptoms suspicious for PE [7]. Main symptoms and features were shortness of breath, chest pain, crackles, infiltration, and cardiomegaly in patients with or without PE (detected or ruled out by multi-slice CT angiography), in our study. Routine blood tests and arterial blood gases (ABG) results were not different between groups. Pneumonia (70%), and acute exacerbation of COPD (5%) were the main diagnosis in the PE negative group. As revealed in previous studies, symptoms and signs, physical examination, conventional radiography, routine laboratory tests, and ABG examination may not reveal or rule out PE in patients with suspected PE, which is supported by our study.

The pregnancy-associated plasma protein-A (PAPP-A) is a matrix metalloproteinase which is primarily synthesized and released to circulation by placental trophoblasts during pregnancy. It is used for screening of Down syndrome in pregnancy [3]. In a study on vascular smooth muscle cells obtained from human coronary arteries, Bayes-Genis et al. demonstrated that vascular smooth muscles cells are able to synthesize and release PAPP-A. Authors also reported that PAPP-A cause plaque progression and destabilization by contributing inflammatory cytokine release from macrophages and LDL-cholesterol accumulation [4]. In the light of these data, several studies have been performed suggesting that PAPP-A, an inflammatory marker like CRP, may be used in both diagnosis and prognostication of acute coronary syndromes [8-12]. It has been thought that PAPP-A might be a marker of unstable plaque rupture in patients with sudden cardiac death [9]. The elevation of PAPP-A may be an independent predictor of ischemic events [10]. There is a weak correlation between PAPP-A and CK-MB; thus, the elevation of PAPP-A may not be related to necrosis. In acute coronary syndromes, it has been proposed that the disruption of plaque stability by proteolytic enzymes released due to local inflammation is accounted from the rupture of atherosclerotic plaques within vessel in acute coronary syndromes [11]. The inflammation is not only local but also systemic [12]. Liuzzo et al. reported that CRP, the prototype marker of inflammation, was elevated in coronary diseases [13]. On the other hand, CRP may increase in many diseases, especially in pneumonia, as it is a non-specific inflammatory marker.

There are some studies investigating whether serum PAPP-A levels are increased in conditions other than pregnancy and atherosclerotic events. Bulut et al. reported that PAPP-A, as an inflammatory marker, was increased in patients with lung cancer [14]. Again, in another study it has been shown that PAPP-A release might play an important role in the development and progression of lung cancer [15].

In another study, it has been reported that serum PAPP-A level was significantly higher in asthmatic patients when compared with healthy controls and there was a correlation between severity of asthma and serum PAPP-A level [16]. Fialova et al. reported that serum PAPP-A levels were increased in chronic renal diseases in relation to oxidative stress and inflammation [17]. In a study investigating serum PAPP-A levels in 1,448 patients with different disorders but not coronary artery disease, no significant elevation was detected in serum PAPP-A levels across any diseases subgroup. In addition, no correlation was detected between serum PAPP-A level and other inflammatory markers such as NT-proBNP, creatinine or CRP. The PAPP-A might potentially be a specific marker for heart diseases but it seems to have lower specificity in patients without coronary heart disease [18]. However, to our knowledge, there is no study evaluating the relation between PE and PAPP-A in English literature.

In our study, PE was confirmed in 24 out of the 53 patients with suspected PE, while it was excluded in 29 patients. Of the patients in whom PE was excluded, about 70% had pneumonia. No significant difference was found in serum CRP and PAPP-A levels between the two groups. This can be explained by the presence of inflammation in both conditions. Although there are no sufficient data in this context, we think that our results are in agreement with the literature in general. Lack of healthy control group may be seen as a limitation. However, it would not be useful in the differential diagnosis of PE, even if serum PAPP-A levels are significantly higher than healthy controls.

## Conclusions

In conclusion, serum PAPP-A level has no significant role either to establish the diagnosis or in differential diagnosis in patients with suspected PE.

## Competing interests

The authors declared no potential conflicts of interest with respect to the research, authorship, and/or publication of this article.
